# Improving genetic diagnosis by disease-specific, ACMG/AMP variant interpretation guidelines for hearing loss

**DOI:** 10.1038/s41598-022-16661-x

**Published:** 2022-07-21

**Authors:** So Young Kim, Bong Jik Kim, Doo Yi Oh, Jin Hee Han, Nayoung Yi, Namju Justin Kim, Moo Kyun Park, Changwon Keum, Go Hun Seo, Byung Yoon Choi

**Affiliations:** 1 Department of Otorhinolaryngology-Head and Neck Surgery, CHA Bundang Medical Center, CHA University, Seongnam, South Korea; 2 Department of Otolaryngology-Head and Neck Surgery, Chungnam National University Sejong Hospital, Chungnam National University College of Medicine, Daejeon, South Korea; 3 Department of Otorhinolaryngology-Head and Neck Surgery, Seoul National University Bundang Hospital, Seongnam, South Korea; 4 Department of Biological Sciences, Vanderbilt University, Nashville, USA; 5 Department of Otorhinolaryngology, Seoul National University College of Medicine, Seoul, South Korea; 6 3Billion, Inc., Seoul, South Korea

**Keywords:** Genetics, Diseases

## Abstract

The 2018 Hearing Loss Expert Panel (HL-EP)-specific guidelines specified from the universal 2015 ACMG/AMP guidelines are proposed to be used in genetic HL, which prompted this study. A genetic HL cohort comprising 135 unrelated probands with available exome sequencing data was established. Overall, 169 variants were prioritized as candidates and interpreted using the 2015 ACMG/AMP and 2018 HL-EP guidelines. Changes in rule application and variant classification between the guidelines were compared. The concordance rate of variant classification of each variant between the guidelines was 71.60%, with significant difference. The proportion of pathogenic variants increased from 13.02% (2015) to 29.59% (2018). Variant classifications of autosomal recessive (AR) variants that previously belonged to VUS or likely pathogenic in the 2015 guidelines were changed toward pathogenic in the 2018 guidelines more frequently than those of autosomal dominant variants (29.17% vs. 6.38%, *P* = 0.005). Stratification of the PM3 and PP1 rules in the 2018 guidelines led to more substantial escalation than that in the 2015 guidelines. We compared the disease-specific guidelines (2018) with the universal guidelines (2015) using real-world data. Owing to the sophistication of case-level data, the HL-specific guidelines have more explicitly classified AR variants toward “likely pathogenic” or “pathogenic”, serving as potential references for other recessive genetic diseases.

## Introduction

The advent of high-throughput next-generation sequencing yielded a significant number of novel variants of unknown pathogenicity and accelerated the discovery of deafness genes^1–3^. However, massive sequencing information given to clinicians and geneticists makes it challenging to decide whether the variant detected through bioinformatics tools is a disease-causing variant. Sometimes, variants that were previously regarded as pathogenic were disqualified later by more evidence-based evaluation as not being related to the disease^4^.

To aid in the classification of all variants in Mendelian disease genes, the American College of Medical Genetics and Genomics (ACMG) and the Association for Molecular Pathology (AMP) proposed Standards and Guidelines for the Interpretation of Sequence Variants in 2015 (2015 ACMG/AMP guidelines), establishing a classification system for sequence variants^5^. Furthermore, each disease entity has its own characteristics in terms of genotypic spectrum, which requires a different approach to confirm the pathogenicity^6^. Interestingly, deafness has high genetic and allelic heterogeneity with a rapidly increasing number of genes, now more than 200 (https://hereditaryhearingloss.org/), which makes the interpretation of novel variants challenging. To provide expert guidance for standardized genomic interpretation in the context of deafness, the Hearing Loss Variant Curation Expert Panel of ACMG/AMP reported the Expert specification of the ACMG/AMP variant interpretation guidelines (2018 HL-EP guidelines) for genetic hearing loss in 2018^7^. Additional branch guidelines for refining the specific rule, such as PVS1 (criterion as very strong) reported in the 2015 original guidelines were also published, helping to interpret each variant^8^.

In the 2018 HL-EP ACMG/AMP guideline, the HL-EP refined the ACMG/AMP guidelines by interpreting their pilot variants comprising a set of 51 variants from major deafness genes. In addition, the HL-EP interpreted a set of 157 variants across nine HL genes using 2018 HL-EP guidelines, which solved the ambiguous classification in 75% (109/157) of variants^9^. They evaluated the impact of their new rule on hearing loss variant specification. However, cautious interpretation is required because the pilot variants in the HL-EP were selected to test as many rule specifications in the 2015 ACMG/AMP guidelines as possible. Therefore, this field has awaited direct comparison of the two guidelines using a real, large cohort with hearing loss. Given this background, our study compared the 2015 and 2018 guidelines using variants from a real, large HL cohort consecutively recruited during a selected period and analyzed whether modifications in the 2018 HL-EP guidelines significantly move VUS toward either benign or pathogenic so that the modification of the rules assists in the accurate diagnosis of genetic hearing loss in a clinical setting. In addition to the comparison of rules and classifications of variants, the Bayesian score was calculated for the 2018 HL-EP and the 2015 ACMG/AMP rules to quantitatively compare the pathogenicity scores of variants.

## Materials and methods

### Ethical considerations

The Institutional Ethical Committee of Seoul National University Bundang Hospital (SNUBH) (IRB-B-1007-105-402) and the Seoul National University Hospital (SNUH) (IRBY-H-0905-041-281) approved the present study. Written informed consent was obtained from the patients or their legal representatives for minors. All study protocols complied with the regulations of the institutional ethical committee of SNUBH and SNUH.

### Variant prioritization

We established a hearing loss cohort comprising 135 genetically unrelated probands with varying degrees of hearing loss, hereafter referred to as the HL cohort. The 169 variants were selected from 135 genetically unrelated probands. All variants were prioritized from ES data, which was conducted as previously described^10,11^. According to the pedigrees, the number of variants in genes with autosomal recessive (AR) inheritance was 120, while that of obvious autosomal dominant (AD) cases was 47. Two variants were categorized as X-linked inheritance. All 135 unrelated probands of the HL cohort underwent exome sequencing (ES). The 169 variants were prioritized as candidate variants for these 135 SNHL patients based on the 2015 ACMG/AMP guidelines for the interpretation of sequence variants^5^.

### Analysis of concordance between the 2015 ACMG/AMP and 2018 HL-EP guidelines

**Figure 1:**
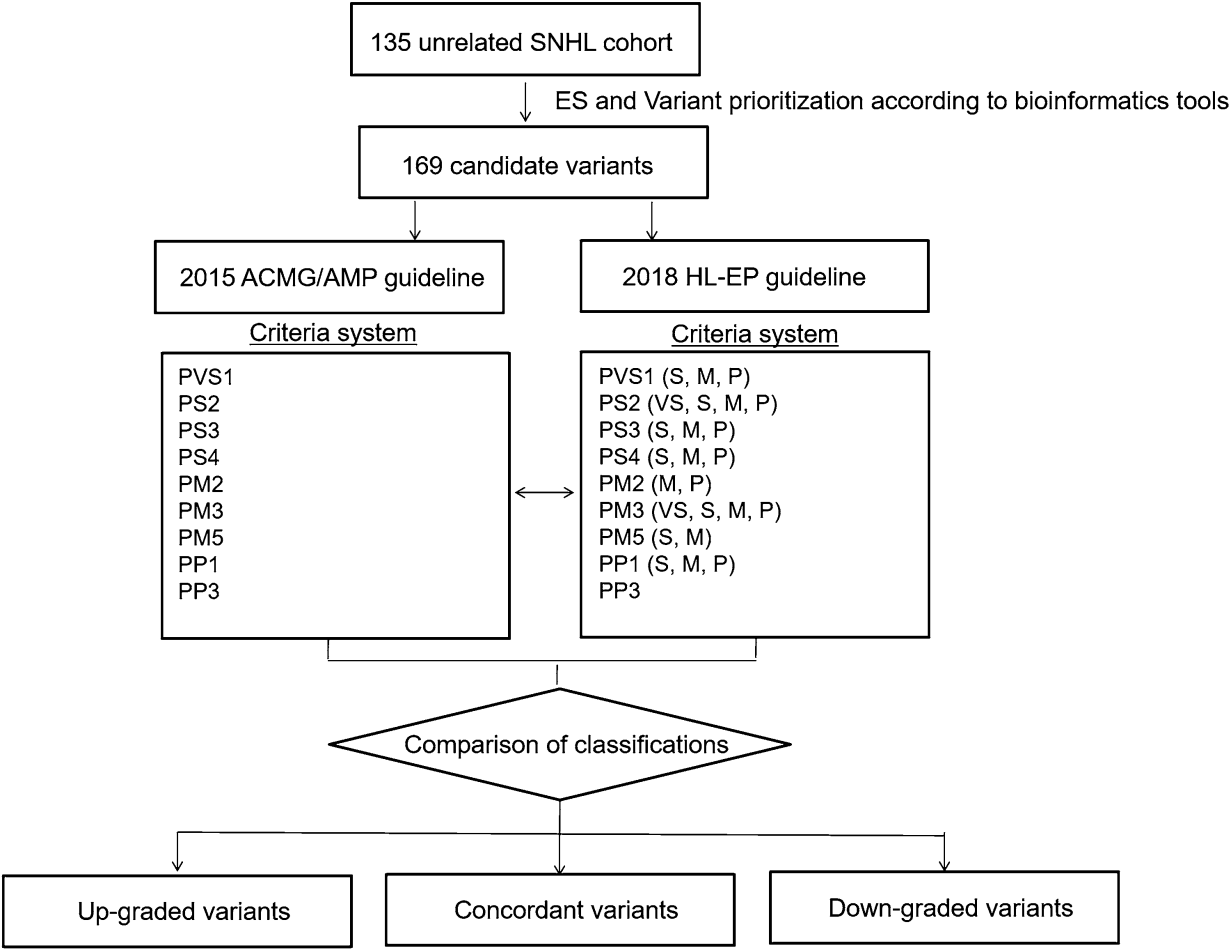
The study design of the present study. The 169 variants of 135 genetically unrelated sensorineural hearing loss probands were classified based on the 2015 ACMG/AMP and 2018 HL-EP guidelines. The variants classification between the 2015 ACMG/AMP and 2018 HL-EP guidelines were compared.

The 169 candidate variants were independently interpreted based on the 2018 HL-EP guidelines^7^ (Fig. 1 and Table S1). The concordance of classifications and criteria for which our variants were eligible were compared between the 2015 ACMG/AMP and 2018 HL-EP guidelines. Variants were classified as pathogenic, likely pathogenic, uncertain significance (VUS), likely benign, or benign^5^. The concordance rate of classification of variants was calculated. For variants with different classifications between the 2015 ACMG/AMP and 2018 HL-EP guidelines, the variants interpreted as lower pathogenic classifications in the 2018 HL-EP guidelines compared to the 2015 ACMG/AMP guidelines were defined as downgraded variants. On the other hand, the variants regarded as higher pathogenic classifications in the 2018 HL-EP guidelines compared to the 2015 ACMG/AMP guidelines were defined as upgraded variants.

The rules applied for variant classification were compared between the two guidelines in a similar manner. The changed rules in the 2018 HL-EP compared to the 2015 ACMG/AMP guidelines included PVS1, PS2, PS3, PM2, PM3, PM5, PP1, and PP3. The PP2 and PP5 rules were excluded in both the 2015 ACMG/AMP guideline and the 2018 HL-EP guidelines. The PVS1 of the 2015 ACMG/AMP guidelines was modified as previously described^8^. Each rule was stratified in more detail according to the degree of pathogenic potential, such as very strong, strong, moderate, and supporting in the specifications of the 2018 HL-EP guidelines. In detail, PS2 was more stratified into very strong, moderate, and supporting. The degrees of moderate and supporting were also added to PS3 and PS4. The highest allele frequency among population databases with a minimum of 2,000 alleles was used to estimate the allele frequencies for PM2, PM2_Supporting, BA1, and BS1. The degree of very strong, strong, and supporting was also available for PM3 depending on the strength of the evidence. Strong and moderate degrees could be applied for the PP1 criteria.

### Comparison of Bayesian values between the 2015 ACMG/AMP and 2018 HL-EP guidelines

The applied rules of the two guidelines were quantified based on Bayesian formulation as previously reported^12^. The transformed Bayesian values for each variant were calculated according to the applied rules of the two guidelines. In brief, the prior probability and odds of pathogenicity were calculated according to the strength and number of each applied rules. Then posterior probability of pathogenicity is estimated based on the formula of Bayes rule using the prior probability and odds of pathogenicity^12^. The distributions of Bayesian values of each variant class were compared between the two guidelines.

## Results

### Comparisons of variant classification

Notably, the distribution of variant classifications was different depending on which of the 2015 ACMG/AMP or 2018 HL-EP guidelines (*P* < 0.001) was applied. Specifically, based on the 2015 ACMG/AMP guidelines, 169 variants from the HL cohort were classified as pathogenic (13.02% [22/169]), likely pathogenic (37.87% [64/169]), and VUS (49.11% [83/169]) (Table 1). However, according to the 2018 HL-EP guidelines, 29.59% (50/169), 23.67% (40/169), 45.56% (77/169), 0.59% (1/169), and 0.59% (1/160) of the candidate variants were classified as pathogenic, likely pathogenic, VUS, likely benign, and benign, respectively. Consistent with this, the concordance rate of variant classification between the 2015 ACMG/AMP and 2018 HL-EP guidelines was 71.60% (121/169). In particular, two guidelines unveiled the most drastic discrepancy in the proportion of ‘likely pathogenic’ variants. In detail, the concordance rates of classification results by the 2015 ACMG/AMP guidelines against those by the 2018 HL-EP guidelines were 95.45% (21/22) for pathogenic variants, 46.88% (30/64) for likely pathogenic variants, and 84.34% (70/83) for VUS. As a result, the proportion of likely pathogenic variants was higher in the 2015 ACMG/AMP guidelines (*P* = 0.007), while that of pathogenic variants was higher in the 2018 HL-EP guidelines (*P* < 0.001).

**Table 1:** Comparisons of variant classifications between 2018 Expert Panel classifications and 2015 ACMG/AMP guideline.

	2018 HL-EP guideline					Total	P-value
	P	LP	VUS	LB	B		
2015 ACMG/AMP							< 0.001*
P	21	1				22 (13.02)	< 0.001**
LP	26	30	7	1		64 (37.87)	0.007**
VUS	3	9	70		1	83 (49.11)	0.586
Total	50 (29.59)	40 (23.67)	77 (45.56)	1 (0.59)	1 (0.59)	169	

*P* pathogenic, *LP* likely pathogenic, *VUS* variant of uncertain significance, *LB* likely benign, *B* benign.

*P < 0.05, Chi-square test, 2015 ACMG/AMP vs. 2018 HL-EP guideline.

**P < 0.05, Chi-square test, 2015 ACMG/AMP vs. 2018 HL-EP guideline for each classification.

Compared to the classifications of the 2015 ACMG/AMP guideline, 22.49% (38/169) of variants were upgraded in the classifications of the 2018 HL-EP guidelines (Table S2). The benefit of these upgrades was primarily presented in ‘likely pathogenic’ variants. Specifically, the 26 likely pathogenic and three VUS variants were upgraded to pathogenic variants in the 2018 HL-EP guidelines (Table S2). Even though nine VUS variants were upgraded to ‘likely pathogenic’ variants in the 2018 HL-EP guideline, the 7 ‘likely pathogenic’ and one ‘pathogenic’ variant were downgraded to VUS and ‘likely pathogenic’ in the 2018 HL-EP guidelines (Table S3). One ‘likely pathogenic’ variant and one VUS were downgraded to a ‘likely benign’ and ‘benign’ variant in the 2018 HL-EP guidelines. The 5.92% (10/169) of variants downgraded in the classifications of the 2018 HL-EP guidelines (Table S3). In terms of moving VUS toward either benign or pathogenic, 13 (15.66%) of 83 VUS variants classified in the 2015 guidelines were reinterpreted as either pathogenic/likely pathogenic (n = 12,14.45%) or benign (n = 1, 1.20%) in the 2018 guidelines (Table 2). Overall, application of the 2018 HL-EP guidelines lead to a significant increase in the proportion of variants with more explicit classification (pathogenic, likely pathogenic, and benign).

**Table 2:** Changes of variant classifications according to inheritance patterns.

Changes	Classifications	Inheritance			Total	P-value
		AD	AR	X-linked		
Un-changed	Concordance	82.98 (39/47)	66.67 (80/120)	100.0 (2/2)	71.60 (121/169)	0.101
Up-graded	VUS → LP	2	7	0	9	
	VUS → P	0	3	0	3	
	LP → P	1	25	0	26	
	Total	6.38 (3/47)	29.17 (35/120)	0 (0/2)	22.49 (38/169)	0.002**
Down-graded	VUS → B	0	1	0	1	
	LP → VUS	4	3	0	7	
	LP → LB	0	1	0	1	
	P → LP	1	0	0	1	
	Total	10.64 (5/47)	4.17 (5/120)	0 (0/2)	5.92 (10/169)	0.146
Total		27.81 (47/169)	71.0 (120/169)	1.2 (2/169)		

*P* pathogenic, *LP* likely pathogenic, *VUS* variant of uncertain significance, *LB* likely benign, *B* benign.

*P < 0.05, Chi-square test, Un-changed variants of AD vs. AR.

**P < 0.05, Chi-square test, Up-graded variants of AD vs. AR.

**Figure 2:**
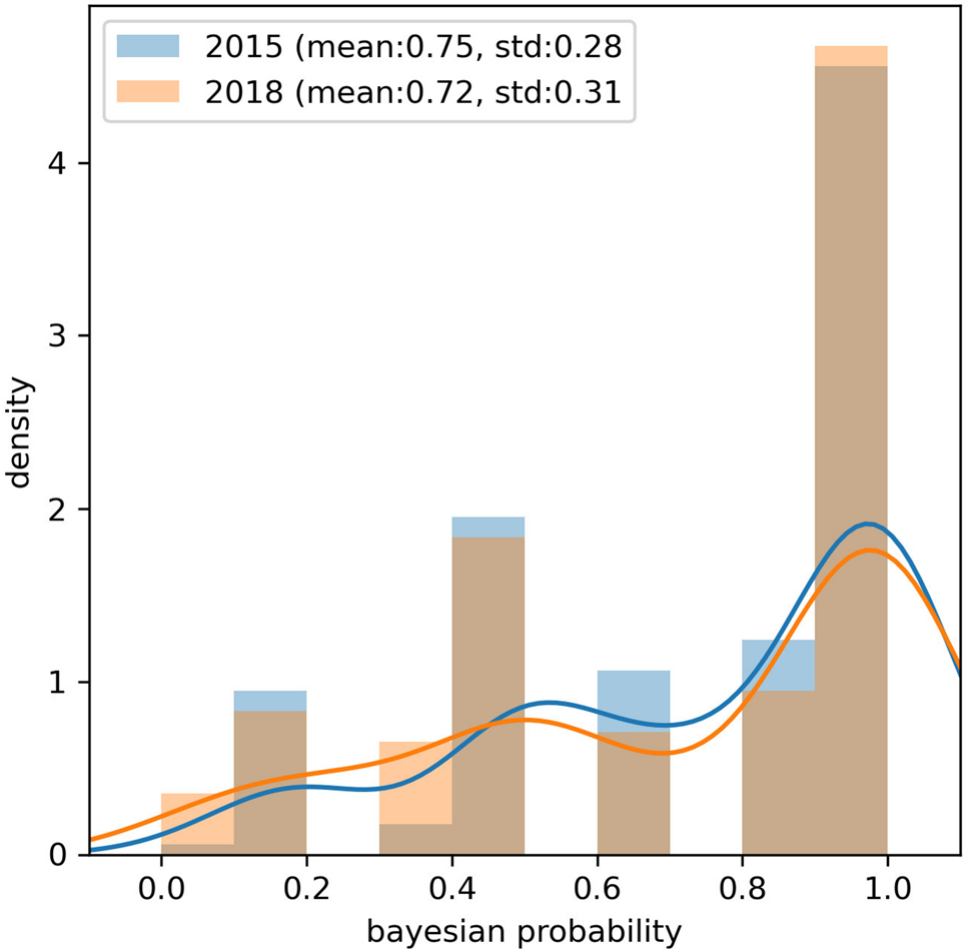
Comparison of Bayesian values between the 2015 ACMG/AMP and 2018 HL-EP guidelines.

The distributions of Bayesian values did not differ between the 2015 ACMG/AMP and 2018 HL-EP guidelines in both overall and each variant classification of pathogenic variants, likely pathogenic variants, and VUS (Fig. 2).

Next, the variants were broken down into the variants in genes with AR, AD, X-linked inheritance. There was difference between the variants in genes with AR and AD inheritances in the proportion of variants that changed in classifications from the 2015 ACMG/AMP to the 2018 HL-EP guidelines (33.33% [40/120] vs. 17.02% [8/47], *p* = 0.038, Table 2). In addition, the rates of upgraded variants were higher for the variants in genes with AR inheritance than for the variants in genes with AD inheritance (29.17% [35/120] vs. 6.38% [3/47], *p* = 0.002), while the number of downgraded variants was not significantly different between the variants in genes with AR and AD inheritances (4.17% [5/120] vs. 10.64% [45/47], *p* = 0.146).

### Overall frequencies of applied criteria

**Figure 3:**
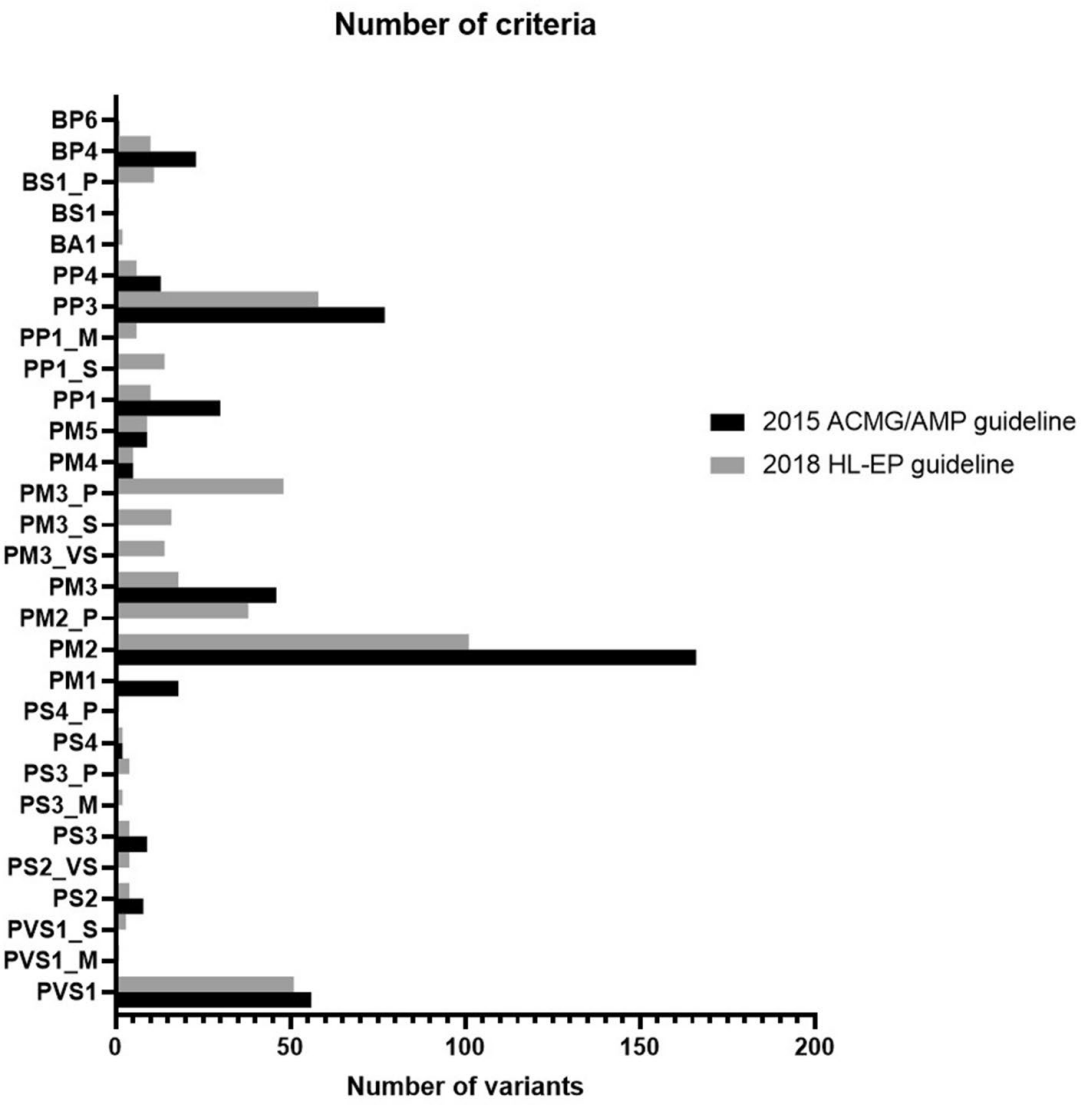
Frequency of criteria applied in the 2015 ACMG/AMP and 2018 HL-EP guidelines. The most commonly applied rule in the two guidelines was PM2 and the criterion of PM3 yielded the most drastic difference between the 2015 ACMG/AMP and 2018 HL-EP guidelines.

The most commonly applied rule in the two guidelines was PM2, which was applied to 166 and 101 variants in the 2015 ACMG/AMP and 2018 HL-EP guidelines, respectively (Fig. 3).

A couple of rules saw significant differences between the guidelines. The criterion of PM3 yielded the most drastic difference between the 2015 ACMG/AMP and 2018 HL-EP guidelines (46 variants vs. 96 variants [PM3 = 18, PM3_VS = 14, PM3_S = 16, and PM3_P = 48]). The criterion of PP1 showed the second largest difference between the 2015 ACMG/AMP and 2018 HL-EP guidelines (31 vs. 10 variants of PP1, 14 variants for PP1_S, and six for PP1_M). The criterion of PM2 showed the third largest difference between the 2015 ACMG/AMP and 2018 HL-EP guidelines (166 vs. 101 variants). As a result, the 2018 HL-EP guidelines resulted in the redistribution of variant classifications from those of the 2015 ACMG/AMP guidelines (*P* < 0.001, Table 1).

### Case-level data (PS2, PS4, PM3, PM6, and PP1)

Criteria evaluating case-level data showed the most significant difference between the two guidelines, which was pronounced especially in PM3 and PP1 rules. Among 46 variants with the PM3 rule in the 2015 ACMG/AMP guidelines, 30.4% (14/46) and 34.8% (16/46) of variants have become newly eligible for higher strength criteria, PM3_VS and PM3_S, respectively, in the 2018 HL-EP guidelines. These changes have led to the upgrade of the variant classification in 28 variants, with six variants from VUS to likely pathogenic, two from VUS to pathogenic, and 20 from likely pathogenic to pathogenic (Table S2).

A total of 30 variants were eligible for the PP1 rule in the 2015 ACMG/AMP guidelines. In the 2018 HL-EP guidelines, these 30 variants were stratified into PP1 (n = 10, 33.3%), PP1_S (n = 14, 46.7%), and PP1_M rules (n = 6, 20.0%). In particular, the change from PP1 to PP1_S has upgraded three variants from VUS to likely pathogenic or pathogenic variants (Table S2).

Eight variants were categorized as PS2 in the 2015 ACMG/AMP guidelines. Among the eight variants eligible for PS2 in the 2015 ACMG/AMP guidelines, four maintained PS2 and the other four variants were upgraded to PS2_VS in the 2018 HL-EP guidelines.

### Population frequency data (PM2, BS1, BS2, and BA1)

The PM2 rule also merits attention because it is the most commonly applied rule of the two guidelines. Indeed, 166 variants were eligible for this rule in the 2015 ACMG/AMP guidelines. Among these 166 PM2 variants, 60.84% (101/166) consistently maintained the PM2 rule in the 2018 HL-EP guidelines. The remaining 22.89% (38/166), 6.63% (11/166), 0.60% (1/166), 1.20% (2/166), and 2.4% (4/166) of variants were downgraded to PM2_P, BS1_P, BS1, BA1, and were even excluded from the PM2 criterion, respectively.

In the current study, the highest allele frequency of subpopulation with 2,000 or more alleles in gnomAD data was used for eligibility of the population rules. One of the two ‘BA1’ variants (*MYO15A*: c.9478C>T and *MPZL2*:c.220C>T), *MPZL2*:c.220C>T, meets the criteria of PVS1, PM3, and PP1_M, thus, was classified as pathogenic in the 2018 HL-EP guidelines. Given this, we propose this variant to be excluded from the PM2 criterion, to avoid accidentally missing important variants, including ethnic-specific founder alleles or mutational hotspots.

### Variant type and location (PVS1, PS1, PM1, PM4, and PP2)

A total of 56 variants were eligible for the PVS1 rule in the 2015 ACMG/AMP guidelines. Among these 56 variants, four were downgraded to either PVS1_M or PVS1_S, and one was excluded from the PVS1 rule according to the 2018 HL-EP guidelines^7^. A variant (*GJB3* c.538C>T) was downgraded from pathogenic to VUS due to disqualification from eligibility of the PVS1 rule based on the 2018 HL-EP guidelines.

Eighteen variants and one variant were used to meet the PM1 rule under the 2015 ACMG/AMP guidelines and the 2018 HL-EP guidelines, respectively. Regarding the 17 variants disqualified from the PM1 rule based on the 2018 HL-EP guidelines, loss of the eligibility for the PM1 rule for three variants led to the downgrading from “likely pathogenic” to VUS.

### Functional, computational prediction, and phenotypic data (PS3, PP3, PP4, and BS3)

In the 2018 HL-EP guidelines, where the functional data criteria become more stringent, five of nine PS3 variants in the 2015 ACMG/AMP guidelines were downgraded in terms of eligibility for application of the PS3 rules, with two variants to PS3_M and the other three to PS3_P. Specifically, the change from PS3 to PS3_P downgraded the classification of a variant (*NLRP3* c.2752C>T) from likely pathogenic to VUS (Table 2).

Among the 77 variants with the PP3 rule in the 2015 ACMG/AMP guidelines, 24.68% (19/77) of variants lost eligibility for the PP3 rule in the 2018 HL-EP guidelines. Notably, three of the 19 variants were downgraded from ‘likely pathogenic’ to ‘VUS’ due to exclusion from both the PP3 and PP4 rules (Table 2).

Thirteen variants were applied to the PP4 rule in the 2015 ACMG/AMP guidelines. Among the 13 variants, only four maintained the PP4 rule in the 2018 HL-EP guidelines based on the specification list of PP4. However, six variants in our cohort had a specific phenotype that could be added to the specification list of PP4 in the 2018 HL-EP guidelines (Table S4).

### The comparisons with the HL-EP interpretations on the 51 pilot variants

The HL-EP interpreted the 51 pilot variants of nine genes using the 2018 HL-EP guidelines. We re-interpreted these pilot variants using the 2015 ACMG/AMP guidelines and compared these results with those of the present cohort (Tables S5 and S6). Seven of the 51 variants (13.7%) showed different variant classifications between the two guidelines. This was a smaller change compared to that of the present study (26.63% [45/169]). However, many of these pilot variants are well-known pathogenic variants (20 pathogenic and seven likely pathogenic variants) or definitively benign variants (three likely benign and 10 benign variants). However, in the clinical condition, more VUS might be identified than the definitely pathogenic or benign variants, as the 75 VUS in our cohort. The present cohort included 169 variants of 51 genes, thereby broadening the applications and comparisons of variant interpretations. Based on this large clinical cohort study, we derived the virtues and several suggestions for the 2018 HL-EP guidelines.

## Discussion

The 2018 HL-EP guidelines changed the variant classes of 28.40% (48/169) variants from those of the 2015 ACMG/AMP guidelines. Although the 2018 HL-EP guidelines could not decrease the portion of VUS compared to those of the 2015 ACMG/AMP guidelines, the 2018 HL-EP guidelines stratified criteria for case-level data (PM3 and PP1). Because both PM3 and PP1 strengthened the criteria of the variants in genes with AR inheritance, the number of upgraded variants was higher in the variants in genes with AR inheritance than in the variants in genes with AD inheritance. On the other hand, the population frequency data, such as PM2 and variant type, and location data, such as PVS1 and PM1, were removed or weakened in the downgraded variants.

The case level criteria of PM3 and PP1 criteria were strengthened in the 2018 HL-EP guidelines compared to those of the 2015 ACMG/AMP guidelines. The potentiation of case-level evidence drew more changes in AR than AD variants, which reflected the efforts of the 2018 HL-EP guidelines to better classify AR variants, because they account for the majority of genetic hearing loss variants. Three upgraded variants in the 51 pilot variants in the 2018 HL-EP guidelines were also the variants in genes with AR inheritance that qualified as PM3_VS (Table S6).

The population frequency criteria (PM2, PM2_P, BS1_P, BS1, and BA1) were more stringently applied in the 2018 HL-EP guidelines than in the 2015 ACMG/AMP guidelines based on the allele frequency cutoff studies^13,14^. These modifications of the population frequency criteria weakened variant classifications. The *GJB3: c.538C*>*T* was downgraded from pathogenic variants (PVS1, PM2, and PP5) to ‘likely benign’ variant (BS1_P, BP4). Although *GJB3* was previously suggested as a deafness gene for high-frequency hearing loss^15^, growing epidemiologic and functional evidence has demonstrated conflicting results^15,16^. In Korea, the MAF of the reference group was as high as approximately 0.09% (3/1722 individuals), which favors the benign potential of this variant. Thus, the weakening of population criteria in this variant was reasonable in the 2018 HL-EP guidelines. However, the selection of population MAF needs to be undertaken with caution to prevent biased variant classification in the 2018 HL-EP guidelines.

The mutational hotspot or founder variants, such as *MPZL2*:c.220C>T, could be misclassified when the highest allele frequency of subpopulation with 2000 or more alleles was applied^17^. To attenuate the possible misclassification of variants, the Clinical Genome Resource Sequence Variant Interpretation Working Group refined the BA1 rule to specify a set of variants which should be exempted from the criteria of MAF > 0.05 in any general continental population dataset of at least 2000 observed alleles^18^. They permitted the suggestion of additional BA1 exception list through web site (https://www.clinicalgenome.org/working-groups/sequence-variant-interpretation/).

The 2018 HL-EP guidelines defined PS3 as the confident functional data derived from the knock-in mouse model. However, functional data, especially unpublished internal laboratory data, could improve the classification of ambiguous variants^19,20^. The ClinGen *PTEN* Expert Panel demonstrated that the addition of internal laboratory data could re-classify VUS or conflicting variants as likely benign, pathogenic, and likely pathogenic variants^21^. In the present cohort, *NLRP3:* c.2752C>T could be assigned to the PS3 rule in the 2015 ACMG/AMP guidelines based on our internal laboratory data and was classified as a likely pathogenic variant. However, the PS3 rule of *NLRP3:* c.2752C>T was replaced by PS3_P, which resulted in the downgrading of the variant to VUS in the 2018 HL-EP guidelines.

The phenotype-specific criterion (PP4) could be considered to be applied to six additional variants, which are not listed in the 2018 HL-EP guidelines. In addition to the specified genes in the 2018 HL-EP guidelines, specific phenotypes with hearing loss can be manifested in *OPA1* with auditory neuropathy spectrum disorder, *ATP1A3* with CAPOS syndrome (cerebellar ataxia, areflexia, pes cavus, optic atrophy, and sensorineural hearing loss)^22^, *NLRP3* with an AD systemic autoinflammatory disease spectrum, termed cryopyrin-associated periodic syndromes^23^, *COL4A4* with Alport syndrome^24^, *PTPN11* with Noonan syndrome^25^, and *EFTUD2* with mandibulofacial dysostosis with microcephaly^26^. On the other hand, the known syndromic hearing loss gene can be manifested solely as hearing loss without syndromic features, in that it cannot be applied to PP4. Although *DIAPH1* was listed as a gene satisfying PP4 in the 2018 HL-EP guidelines, our probands with *DIAPH1* variants (*DIAPH1:c.793G*>*T* and *DIAPH1:c.2156C*>*T*) did not show macrothrombocytopenia^27^. In summary, the application of PP4 should be flexible to accommodate the newly added syndromic or non-syndromic features.

The stratifications and specifications of the 2018 HL-EP guidelines enhanced the pathogenic variant classifications of hearing loss without the assistance of predicting rules, such as PP2 and PP5. The necessity of PP2 and PP5 is controversial^28,29^. These two rules were removed from both the 2015 ACMG/AMP guidelines and the 2018 HL-EP guidelines in our cohort.

## Limitations of this study

This study evaluated as many as 169 variants from 135 unrelated SNHL cohorts. Based on the fact that about 60% of SNHL is genetic hearing loss, the required size for the study population was calculated to be 52, when it was assumed that effect size was 0.6 with α of 0.05 and power of 0.95 in G power analysis^30^. However, a few limitations should be considered when interpreting the current results. First, the information from the literature and database, such as segregation results was added for some variants, which could have influenced the current variant classifications. To minimize the bias from the available database, the latest updated versions of data were applied and multiple rounds of panel discussions were conducted to finalize the variant classifications. In addition, an automated prioritization system verified the final variant classification results^31^. Secondly, although 169 variants were classified in the present study, the variants were not suitable for evaluating all rules. Therefore, the number of variants for evaluating certain rules was still insufficient and higher number of variants were still needed. Last but not least, this study is based on the Korean ethnic population, thus the application of the 2018 HL-EP guidelines could have been influenced by the Asian background. Because the 2018 HL-EP guideline set the strict population frequency criteria, the ethnic difference of MAF may have affected the current results^32^. Future research with the higher number of variants of genetic hearing loss in other ethnic groups could complement the current limitations.

## Conclusion

The 2018 HL-EP guidelines did not reduce the VUS compared to the general 2015 ACMG/AMP guidelines. However, they changed the number of variants in genes with AR inheritance classifications toward likely pathogenic or pathogenic variants by enhancing the case-level evidence, which fitted the characteristics of inheritance patterns of the deafness genes, where AR inheritance predominates. These specifications of case-level evidence could compensate for the loss of predictive criteria, such as PP5, in the 2018 HL-EP guidelines, which made the guidelines more refined to determine the true causative variant in an elaborate way.

## Supplementary Information

Supplementary Table S1.

Supplementary Tables.

Supplementary Table S6.

## Data Availability

The ES data sets used for the study are available from the corresponding author upon request.
