# Supplementary material for: Improving genetic diagnosis by disease-specific, ACMG/AMP variant interpretation guidelines for hearing loss
Source: Sci Rep. 2022 Jul 21;12:12457. doi: 10.1038/s41598-022-16661-x (PMC9304357; doi:10.1038/s41598-022-16661-x)
Supplement: Supplementary file 2 — Supplementary Tables. [file 41598_2022_16661_MOESM2_ESM.docx]

**Table S2** Variants classified as up-graded in the 2018 HL-EP guideline compared to the 2015 ACMG/AMP guideline (N = 38)

| Gene | Nucleotide Change | Protein Change | Position | 2015 Classification | 2015 Criteria | 2018 Classification | 2018 Criteria |
| --- | --- | --- | --- | --- | --- | --- | --- |
| *MYO7A* | NM_000260.4:c.721C>G | NP_000251:p.Arg241Gly | Chr11:76868036 | LP | PM1, PM2, PM5, PP1, PP3 | P | PM2, PM3_VS, PM5, PP1_S, PP3 |
| *MYO7A* | NC_000011.9(NM_000260.3):c.2695-9A>G | Frameshif, PMID: 20052763 | Chr11:76892417 | VUS | PM2, PP3 | P | PVS1_S, PM2, PM3_S, PP3 |
| *OTOF* | NM_001287489.1:c.2521G>A | NP_919224.1:p.Glu841Lys | Chr2:26700042 | LP | PM2, PM3, PP3, PP4 | P | PM2, PM3_VS, PP3 |
| *MYO15A* | NM_016239.3:c.900delT | NP_057323.3:p.Asp300fs | Chr17:18023014 | LP | PVS1, PM2 | P | PVS1, PM2, PM3_P |
| *OTOF* | NM_001287489.2:c.3032T>C | NP_001274418:p.Leu1011Pro | Chr2:26698321 | LP | PM2, PM3, PP1, PP3, PP4 | P | PM2, PM3_VS, PP1, PP3 |
| *TMC1* | NM_138691.3:c.1714G>A | NP_619636:p.Asp572Asn | Chr9:75431077 | VUS | PM2, PP1, BP4 | LP | PM2, PP1_S |
| *DFNA5* | NC_000007.13(NM_004403.2):c.991-15_991-13delTTC |  | Chr7:24746008 | VUS | PM2, PP1, PP3 | LP | PM2, PP1_S, PP3 |
| *CDH23* | NM_022124.6:c.719C>T | NP_071407:p.Pro240Leu | Chr10:73330641 | LP | PM1, PM2, PM3, PP1, PP3 | P | PM1, PM2, PM3_VS, PP1_M |
| *STRC* | NM_153700.2:c.5141_5142delTG | NP_714544:p.Val1714Glyfs*5 | Chr15:43892255 | LP | PVS1, PM2 | P | PVS1, PM2, PM3_P |
| *STRC* | NM_153700.2:c.4816dupC | NP_714544:p.Leu1606Profs*25 | Chr15:43893097 | LP | PVS1, PM2 | P | PVS1, PM2, PM3_P |
| *STRC* | NM_153700.2:c.4057C>T | NP_714544:p.Gln1353* | Chr15:43896918 | LP | PVS1, PM2 | P | PVS1, PM2, PM3_S |
| *CDH23* | NM_022124.5:c.3076delC | NP_071407:Leu1026Serfs*27 | Chr10:73466775 | LP | PVS1, PM2 | P | PVS1, PM2, PM3 |
| *CLCNKB* | NM_001165945.2:c.118delA | NP_001159417:p.Arg40Glyfs*4 | Chr1:16375584 | LP | PVS1, PM2 | P | PVS1, PM2_P, PM3_P |
| *CLCNKB* | NM_000085.4:c.1166G>A | NP_000076:p.Trp389* | Chr1:16377482 | LP | PVS1, PM2 | P | PVS1, PM2, PM3_P |
| *MYO7A* | NC_000011.9(NM_000260.3):c.2095-2A>T |  | Chr11:76886416 | LP | PVS1, PM2 | P | PVS1, PM2, PM3_P |
| *MYO7A* | NM_000260.3:c.6487G>A | NP_000251:p.Gly2163Ser | Chr11:76924953 | VUS | PM2, PP1, PP3 | P | PM2, PM3_P, PP1_S |
| *USH2A* | NM_206933.2:c.14835delT | NP_996816:p.Val4946Trpfs*4 | Chr1:215814032 | LP | PVS1, PM2 | P | PVS1, PM2, PM3_P |
| *USH2A* | NM_206933.2:c.13112_13115delAAAT | NP_996816:p.Gln4371Argfs*19 | Chr1:215848137 | LP | PVS1, PM2 | P | PVS1, PM2, PM3_P |
| *GJB2* | NM_004004.5:c.299_300del | NP_003995:p.His100Argfs*14 | Chr13:20763420 | LP | PVS1, PM2 | P | PVS1, PM2, PM3_S |
| *OTOF* | NM_001287489.1:c.5566C>T | NP_001274418:p.Arg1856Trp | Chr2:26683866 | VUS | PM2, PM3, PP3 | LP | PM2, PM3_S, PP3, PP4 |
| *SLC26A4* | NM_000441.1:c.2168A>G | NP_000432:p.His723Arg | Chr7:107350577 | LP | PM1, PM2, PM3, PP3 | P | PM2_P, PM3_VS, PS1, PP3 |
| *TRIOBP* | NM_001039141.2:c.874dupG | NP_001034230:p.Ala292fs | Chr22:38119434 | LP | PVS1, PM2 | P | PVS1, PM2, PM3_P |
| *TRIOBP* | NM_001039141.2:c.1195C>T | NP_001034230:p.Arg399* | Chr22:38119758 | LP | PVS1, PM2 | P | PVS1, PM2, PM3_P |
| *OTOG* | NM_001277269.1:c.330C>G | NP_001264198:p.Tyr110* | Chr11:17574667 | LP | PVS1, PM2 | P | PVS1, PM2, PM3_P |
| *OTOG* | NM_001277269.1:c.2207delA | NP_001264198:p.Gln736Argfs*79 | Chr11:17594714 | LP | PVS1, PM2 | P | PVS1, PM2, PM3_P |
| *GJB2* | NM_004004.5:c.427C>T | NP_003995:p.Arg143Trp | Chr13:20763294 | LP | PM1, PM2, PM3, PM5, PP3 | P | PM2_P, PM3_VS, PM5, PP3 |
| *GJB2* | NM_004004.5:c.109G>A | NP_003995:p.Val37Ile | Chr13:20763612 | LP | PM1, PM5, PP1, PP3 | P | PS4, PM3, PM5, PP1_S |
| *COL4A4* | NM_000092.4:c.1323_1340delTGGCTTGCCTGGAGCACC | NP_000083:p.Gly442_Pro447del | Chr2:227958869 | VUS | PM2, PM4 | LP | PM2_P, PM3_S, PM4 |
| *MYO15A* | NM_016239.3: c.10250_10252delCCT | NP_057323.3:p.Ser3417del | Chr17:18052868 / Chr17:18075498 | LP | PM2, PM3, PM4, PP1 | P | PM2, PM3_S, PM4, PP1_M |
| *USH2A* | NM_206933.2:c.2802T>G | NP_996816:p.Cys934Trp | Chr1:216419934 | VUS | PM2, PM3, PP3 | P | PM3_VS, PM2_P, PP3 |
| *MYO15A* | NM_016239.3:c.8183G>A | NP_057323.3:p.Arg2728His | Chr17:18058028 | VUS | PM2, PM3, PP3 | LP | PM3_S, PM2_P, PP3 |
| *LOXHD1* | NC_000018.9(NM_001145472.2):c.1938+5G>C |  | Chr18:44101055 | VUS | PM2, PM3, PP1, BP4 | LP | PS3_P, PM2, PM3, PP1_M |
| *MYO15A* | NM_016239.3:c.4666G>A | NP_057323.3:p.Ala1556Thr | Chr17:18039887 | VUS | PM2, PM3 | LP | PM2, PM3_S, PP3 |
| *PTPN11* | NM_002834.3:c.922A>G | NP_002825.3:p.Asn308Asp | Chr12:112915523 | LP | PS2, PM2, PM5, PP1, PP3 | P | PS2_VS, PS3, PM1, PM2, PM5, PP1_S, PP3 |
| *TMPRSS3* | NM_024022.2:c.771C>G | NP_076927:p.His257Gln | Chr21:43803153 | VUS | PM2, PM3, PP3 | LP | PM2, PM3_S, PP3 |
| *TMPRSS3* | NM_024022.2:c.743C>T | NP_076927:p.Thr248Met | Chr21:43803181 | VUS | PM2, PM3, PP3 | LP | PM2, PM3_S, PP3 |
| *TMPRSS3* | NM_024022.2:c.916G>A | NP_076927:p.Ala306Thr | Chr21:43802210 | LP | PM1, PM2, PM3, PP1,PP3 | P | PM2_P, PM3_VS, PP1_S, PP3 |
| *TMPRSS3* | NM_024022.2:c.325C>T | NP_076927:p.Arg109Trp | Chr21:43808633 | LP | PS3, PM1, PM2, PP3 | P | PS3, PM2_P, PM3_VS, PP3 |

P: Pathogenic, LP: Likely pathogenic, VUS: Variant of uncertain significance

**Table S3** Variants classified down-graded in the 2018 HL-EP guideline compared to the 2015 ACMG/AMP guideline (N = 10)

| Gene | Nucleotide Change | Protein Change | Position | 2015 Classification | 2015 Criteria | 2018 Classification | 2018 Criteria |
| --- | --- | --- | --- | --- | --- | --- | --- |
| *OPA1* | NM_130837.2:c.1057A>C | NP_570850:p.Ser353Arg | Chr3:193355762 | LP | PM1, PM2, PP3, PP4 | VUS | PM2, PP3 |
| *TRIOBP* | NC_000022.10(NM_001039141.2):c.-60-2A>C | | Chr22:38097311 | LP | PVS1, PM2 | VUS | PVS1, BS1 |
| *MYO6* | NC_000006.11(NM_004999.3):c.3137+1G>A | | Chr6:76604978 | LP | PVS1, PM2 | VUS | PVS1 |
| *OTOA* | NC_000016.9(NM_144672.3):c.1320+5G>C | | Chr16:21721429 | LP | PS3, PM2, PM3, PP3 | VUS | PS3_P, PM2_P, PM3, PP3 |
| *GRHL2* | NM_024915.3:c.1609C>T | NP_079191:p.Arg537* | Chr8:102656450 | LP | PVS1, PM2 | VUS | PVS1 |
| *NLRP3* | NM_001243133.1:c.2752C>T | NP_001230062:p.Arg918* | Chr1:247607362 | LP | PS3, PM1, PM2 | VUS | PM2, PS3_P, PS4_P |
| *USH2A* | NM_206933.2:c.8232G>C | NP_996816:p.Trp2744Cys | Chr1:216052432 | LP | PM2, PM3, PP3, PP4 | VUS | PM2_P, PM3 |
| *GJB3* | NM_024009.2:c.538C>T | NP_076872.1:p.Arg180Ter | Chr1:35250901 | LP | PVS1, PM2, BP4 | LB | BS1_P, BP4 |
| *MYO15A* | NM_016239.4:c.9478C>T | NP_057323.3:p.Leu3160Phe | Chr17:18064722 | VUS | PP3, BP6 | Benign | PP3, BA1 |
| *PTPN11* | NM_002834.4:c.1510A>G | NP_002825.3:p.Met504Val | Chr12:112926890 | P | PS2, PS3, PS4, PM1, PM2, PP3 | LP | PS2, PS3_M, PM2, PP3 |

P: Pathogenic, LP: Likely pathogenic, VUS: Variant of uncertain significance, LB: Likely benign, B: benign

**Table S4** Proposed variants categorized as PP4 in the 2018 HL-EP guideline (N = 6)

| Gene | Nucleotide Change | Protein Change | Position | 2015 ACMG/AMP guideline | | 2018 HL-EP guideline | |
| --- | --- | --- | --- | --- | --- | --- | --- |
|  |  |  |  | Classification | Criteria | Classification | Criteria |
| *OPA1* | NM_130837.2:c.1057A>C | NP_570850:p.Ser353Arg | Chr3:193355762 | LP | PM1, PM2, PP3, PP4 | VUS | PM2, PP3 |
| *ATP1A3* | NM_001256214.2:c.2491G>A | NP_001243143:p.Glu831Lys | Chr19:42474427 | P | PS2, PM1, PM2, PP1, PP3, PP4 | P | PS2_VS, PM1, PM2, PP1_S, PP3 |
| *NLRP3* | NM_001243133.1:c.1076T>C | NP_001230062:p.Leu359Ser | Chr1:247587827 | VUS | PM2, PP3, PP4 | VUS | PM2 |
| *COL4A4* | NC_000002.11(NM_000092.4):c.4333+3A>G | | Chr2:227876894 | VUS | PM2, PP3, PP4 | VUS | PM2, PM3_P, PP3 |
| *PTPN11* | NM_002834.3:c.417G>C | NP_002825.3:p.Glu139Asp | Chr12:112891083 | P | PS1, PS2, PS3, PS4, PM2, PP3, PP4 | P | PS2_VS, PS1, PS3, PS4, PM2, PP3 |
| *EFTUD2* | NC_000017.10(NM_001258353.1):c.271+1G>A | NP_001245282.1:p.Glu91Aspfs*24 | Chr17:42963952 | P | PVS1, PS2, PS3, PM2, PP4 | P | PSV1, PS2, PM2, PS3_P |

P: Pathogenic, LP: Likely pathogenic, VUS: Variant of uncertain significance, LB: Likely benign, B: benign

**Table S5** Comparison with a previous study (Oza et al. 2018)

|  |  | Oza et al. 2018 | Present study |
| --- | --- | --- | --- |
| No. of variants |  | 51 | 169 |
| No. of genes |  | 9 | 51 |
| Classifications | Pathogenic | 20 | 50 |
|  | Likely pathogenic | 7 | 43 |
|  | VUS | 11 | 75 |
|  | Likely benign | 3 | 0 |
|  | Benign | 10 | 1 |
| Up-graded | VUS→LP | 0 | 9 |
|  | VUS→P | 0 | 3 |
|  | LP→P | 3 | 26 |
| Down-graded | P→LP | 2 | 1 |
|  | LP→VUS | 0 | 5 |
|  | P→VUS | 0 | 0 |
|  | VUS→B | 1 | 1 |
|  | LB→B | 1 | 0 |
| Changes | Total | 7 (13.7%) | 45 (26.63%) |
| Population criteria | PM2 | 27 | 147 |
|  | PM2_P | 13 | 15 |
|  | BS1_P | 1 | 0 |
|  | BS1 | 7 (5 exclusion) | 0 |
|  | BA1 | 15 (4 exclusion) | 3 (2 exclusion) |
| PM3 criteria | PM3 | 7 | 17 |
|  | PM3_S | 3 | 13 |
|  | PM3_VS | 14 | 14 |
|  | PM3_P | 2 | 49 |

P: Pathogenic, LP: Likely pathogenic, VUS: Variant of uncertain significance, LB: Likely benign, B: benign
